# Multidisciplinary Management of Complex Regional Pain Syndrome (CRPS) Type 1 in the Hand and Wrist: A Case Report

**DOI:** 10.7759/cureus.37227

**Published:** 2023-04-06

**Authors:** Christian DeDi, Micah Jones, Konstantinos Oikonomou, Mallory D Jengo

**Affiliations:** 1 Orthopedic Surgery, Edward Via College of Osteopathic Medicine, Blacksburg, USA; 2 Orthopedics, Edward Via College of Osteopathic Medicine, Blacksburg, USA; 3 Orthopedic Surgery, LewisGale Medical Center, Salem, USA

**Keywords:** delayed diagnosis, complex regional pain, multi-modality pain management, upper extremity trauma, carpal bones

## Abstract

Complex regional pain syndrome (CRPS) is a rare disorder that presents as a highly variable combination of intense regional pain, autonomic and vasomotor disturbances that are uncharacteristic of the inciting trauma or event. We report a 36-year-old male construction worker who presented to the orthopedic department status post crush injury to his hand, with acutely increasing right-hand pain, swelling, skin/hair changes, and dysfunction. Presentation changed over a course of 2-8 weeks, with CRPS becoming the eventual working diagnosis. Initial diagnoses were not made by occupational med, nor the urgent care, and definitive diagnosis was achieved in the orthopedic hand office via a thorough history and physical exam as well as imaging modalities including X-ray, CT, and MRI. A multidisciplinary approach involving aggressive hand therapy, anti-inflammatory agents, high-dose prednisone, Gabapentin, and over-the-counter vitamins and supplements was used in the treatment of this patient. This patient had a unique progression of his condition with respect to his carpus, demonstrating acute reduction of bone density on plain film. Stiffness ensued. This patient’s condition was almost “missed” by the masking of the ulnar ossicle variant (os triangulare), and anatomical snuffbox pain on exam, in the face of initially “normal” X-rays. It is important for providers to recognize the *clinical* signs of complex regional pain syndrome, especially in the acute phase of crush injury, swelling, skin and hair changes, and stiffness, and to treat patients' symptoms with a variety of treatment options due to the marked variability of this condition. The patient has made a favorable recovery with some residual functional deficits, however, the patient stated that his quality of life has been restored despite his current stiffness.

## Introduction

Complex regional pain syndrome (CRPS) is a poorly understood disorder characterized by regional pain that is out of proportion to the presenting trauma or abnormal compared to the usual course of a given trauma [1]. This disorder typically follows a specific traumatic event in the patient's history and there are often associated autonomic and vasomotor abnormalities with extremely variable presentations [2]. CRPS is subdivided into type 1 and type 2. Type 1, previously known as reflex sympathetic dystrophy, is a form of CRPS that is not associated with evidence of peripheral nerve damage. Type 2, also known as causalgia, is a form of CRPS that presents with specific damage to a peripheral nerve [2]. Type 1 is the predominant form making up 90% of CRPS patients. The clinical progression of CRPS is highly variable, however, it is broken up into stages. Stage 1 presents as localized pain, aching, burning, and edema that does not follow a specific dermatome. Hypersensitivity to cold or touch is common as well as vasomotor abnormalities that result in color and temperature changes of the impacted area. Stage 2 is characterized by skin and soft tissue changes that include atrophy, thickening of articular surfaces, hair growth, and development of brawny skin. Stage 2 may also show demineralization and regional necrosis of bone on radiographs. Stage 3 is marked by severe limitations in movement, brittle nails, contracture of digits, and advanced, acute regional necrosis of bone leading to decreased density [1].

## Case presentation

**Figure 1:**
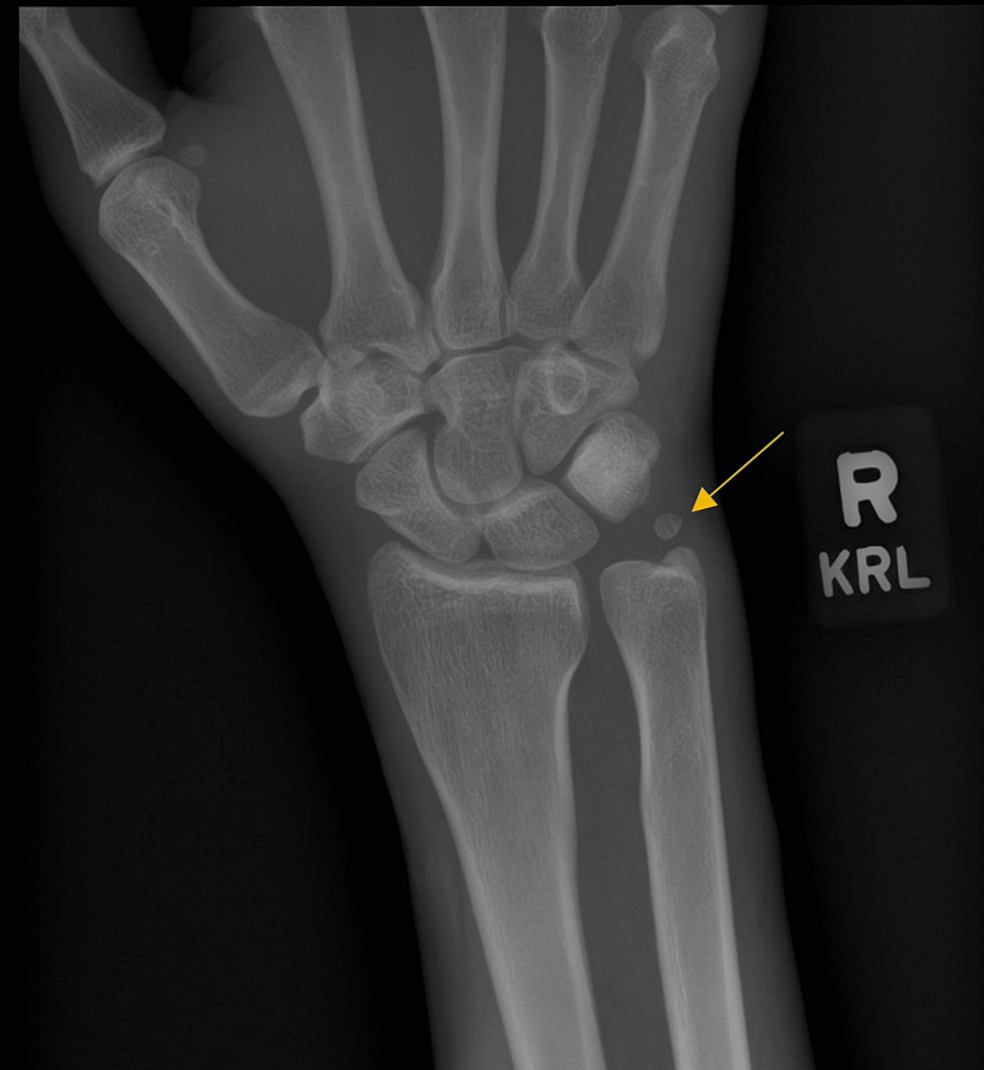
Initial X-ray of the Right Wrist No acute bony abnormalities or soft tissue edema noted. Presence of an ulnar ossicle variant known as an os triangular.

We present a 36-year-old right-hand dominant male who arrived at an orthopedic urgent care clinic complaining of right-hand pain following a work-related injury two days prior. Moderate swelling and ecchymosis were noted on the dorsal aspect of the right hand and wrist at first without any obvious deformities or skin deterioration. The patient was unable to approximate fingers to the palm due to swelling in the hand. There was no appreciable malrotation of the digits and the patient was able to extend all digits completely. The patient was able to actively flex and extend the right wrist, however, the active range of motion is limited by exacerbation of tenderness along the dorsal right hand and metacarpals. There was no current tenderness of the dorsal wrist joint or distal forearm. X-rays of the right wrist and hand were able to identify an anatomic ulnar ossicle variant known as an os triangular with no other acute bony abnormalities (Figure 1). The patient was sent home with an initial diagnosis of right-hand contusion and was instructed to follow up with the hand surgeon’s office a week later for further assessment. Naproxen 500 mg prn and tramadol HCl 50 mg prn were prescribed to the patient for pain management as well as a volar resting splint to wear.

The patient returned to the orthopedic clinic for follow-up by one of our physician assistants nine days after the injury. The patient stated that the medications have provided a small amount of relief and that the pain is still present on the dorsal side of the hand as well as the anatomic snuff box. Physical examination revealed swelling in the dorsum of the hand with tenderness over the anatomic snuff box. Flexor and extensor tendons were all intact with no deformities and no notable abnormalities of the overlying skin. Tenderness was absent at the elbow with supination and pronation. Sensation to light touch was intact with good distal pulses in the right upper extremity. Three views of the right wrist on X-ray did not show evidence of a scaphoid fracture or any other bony abnormalities. The patient was sent home with instruction to continue the naproxen, tramadol, and the volar wrist splint with a thumb spica component. The patient was also instructed to return in a week for a splint off re-exam.

The patient was 16 days from the date of injury at the second follow-up appointment. The pain in the patient's hand was almost completely resolved. Swelling on the dorsum of the hand had reduced as well. Flexors and extensor tendons of the right hand were all intact, however, the patient had an increase in significant stiffness that had developed in his hand and wrist. He also was developing increased hair on the dorsum of his hand, whereby he had to shave his hand secondary to the increased hair formation at one point.

There was no longer tenderness over the anatomic snuffbox or elbow. No gross deformities were noted and the overlying skin was slightly glossy and still swollen. Sensation to light touch and distal pulses were both present. The patient was started on a regimen of oral prednisone pack (Medrol Dosepak) in addition to tramadol. The patient had discontinued naproxen as he felt it was no longer needed. The patient was also instructed to attend aggressive hand therapy in order to restore full flexion and extension of his right hand and wrist. The patient was to follow up in four weeks.

Upon return to the orthopedic clinic, the patient had completed the prednisone and had been participating in hand therapy for three weeks. The patient is now over a month and a half from his original date of injury. Occupational therapy (OT) had helped with pain management even further, although there was a concern for the progression of the swelling and stiffness in his right hand that had extended farther into his wrist joint. Upon physical examination, the patient exhibited a marked reduction in his passive and active arc of motion in his right wrist and hand. There was no point tenderness on the dorsum of the hand or the anatomic snuff box. Sensation to light touch and distal pulses were still intact as well. Mild swelling was noted throughout the hand and wrist without any ecchymosis or erythema. The attending hand surgeon instructed his PA to order an MRI without IV contrast of the right wrist. The patient was to follow up with our hand surgeon for the next visit for further evaluation of atypical healing/progression of the injury and continued concern for working regional osteoporosis and necrosis of the carpal bones.

**Figure 2:**
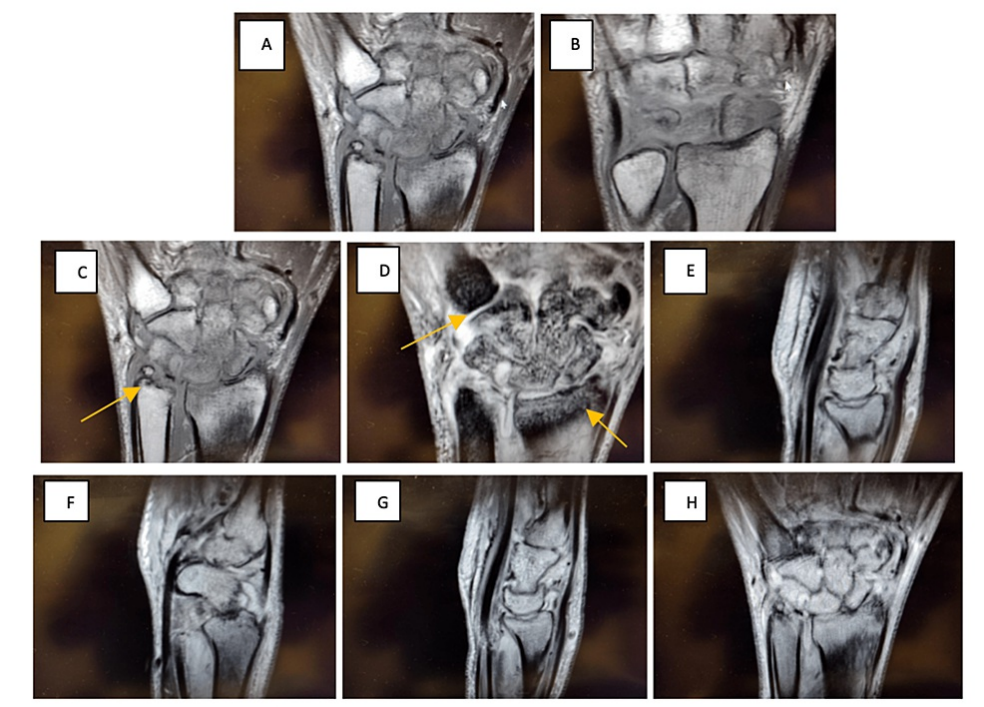
Magnetic Resonance Imaging of the Right Wrist Without Contrast Taken on 06/28/2022 Extensive and recent bony contusions involving all the carpal bones and to a lesser extent the bases of the second and third metacarpals and the distal radius. No discrete fractures or significant intrinsic ligament injury of the wrist is appreciated. Edema involving the intrinsic musculature of the hand, particularly the thenar eminence suggestive of recent muscular contusion. No soft tissue hematoma is appreciated. Probable chronic degenerative perforation of the central disc of the triangular fibrocartilage. The remainder of the right wrist and hand appears otherwise essentially unremarkable.

The patient returned to the clinic for further evaluation by our hand surgeon. His MRI of the right wrist (Figure 2) demonstrated joint space alterations, regional mottling, and edema involving all the carpal bones and to a lesser extent the bases of the second and third metacarpals and the distal radius. No fracture or ligamentous injury of the wrist was present. Edema involving the intrinsic musculature of the hand on the thenar eminence was suggestive of recent muscular contusion. No hematoma. MRI shows a normal ulnar ossicle variant.

The patient had been participating in occupational therapy for one month which helped with pain management and the ability to participate in activities of daily living. The patient practiced a range of motion exercises at home as well. He continued to endorse swelling, poor range of motion, and stiffness in the hand and wrist. He had been wearing a glove to provide compression. He did not feel the course of prednisone provided relief of his stiffness, despite his swelling and acute skin and hair changing being reduced. He had to shave the dorsal aspect of his right hand based on excess hair growth. Upon physical examination, the patient exhibited stiffness of the right wrist as well as the right hand 2nd, 3rd, and 4th metacarpophalangeal (MCP) joints, and proximal interphalangeal (PIP) joints. He lacked full passive motion and mild palpable pain globally. Mild to moderate swelling was noted compared to the left side in addition to an increase in hair on the dorsum of his hands and fingers. He had no significant hypersensitivity. Watson’s test was negative. There was no pain around the triangular fibrocartilage complex (TFCC) and no pain with supination. Sensation was intact. Three views of the right hand on X-ray revealed changes from previous X-rays, demonstrating mottling and absorption of the proximal row of the carpus as well as the capitate. There appeared to be ongoing edema and changes of the bone morphology consistent with bony regional bone necrosis and advanced CRPS.

**Figure 3:**
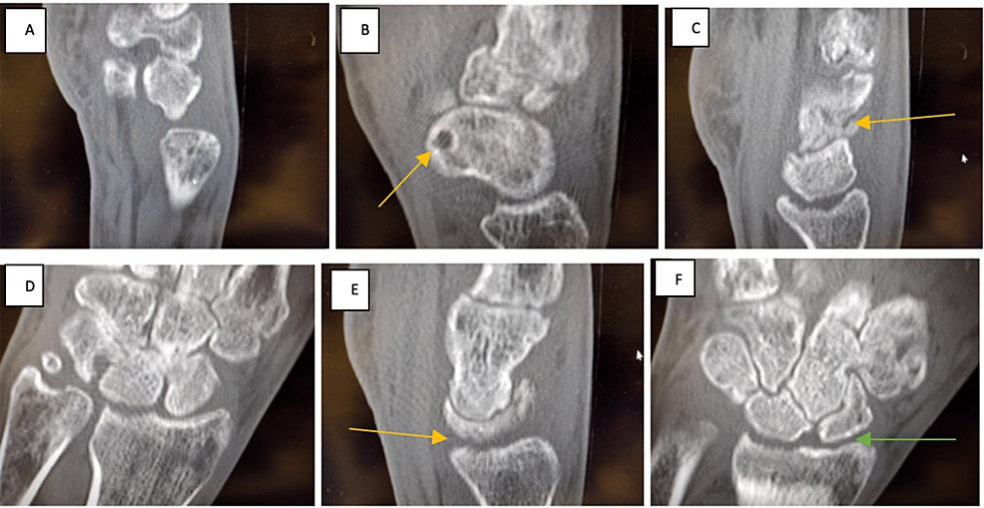
CT Right Wrist Without Contrast Taken on 08/09/2022 Progressive and rather profound osteopenia involving the carpal bones with erosive changes involving the distal radial articular surface with narrowing of the intercarpal joints. The differential includes an erosive arthropathy, possible infective arthritis and/or complex regional pain syndrome. I favor the latter given the history of prior trauma and the imaging findings. No fractures are appreciated.

The patient was educated on the new diagnosis of complex regional pain syndrome type 1. The patient was fitted with a new glove and recommended to continue with aggressive hand exercises in therapy and at home. He was initiated on vitamin B6 200 mg daily and vitamin C 500 mg daily to promote nerve health and provide antioxidant support respectively. He was additionally initiated on a longer and stronger dose of prednisone, starting at 50 mg per day for five days, and tapering down over the course of three weeks, along with daily celecoxib, and gabapentin. The celecoxib was discontinued by the patient as he did not want to take too many medications. A wrist range of motion brace was ordered at this time. There was consideration for manipulation under anesthesia of the MCP and PIP joints if the stiffness persisted. Due to the X-ray revealing wrist derangement, a CT was ordered (Figure 3) for further evaluation. The patient was to follow up in six weeks for repeat X-rays and continue further evaluation.

**Figure 4:**
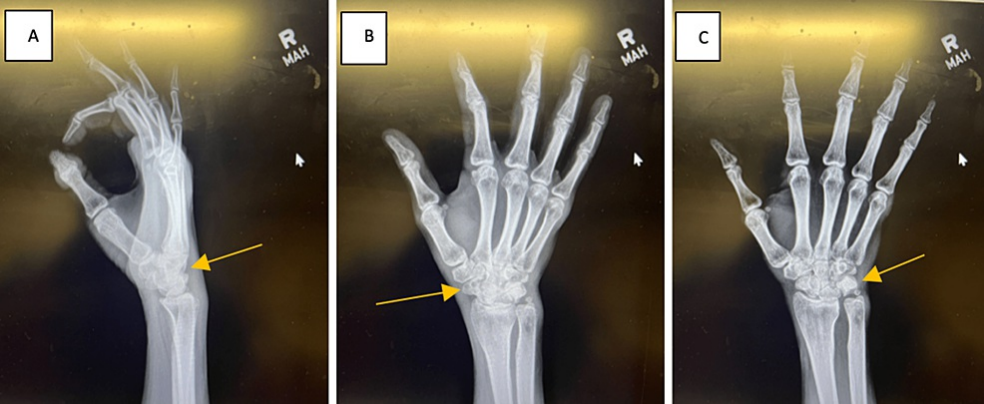
X-rays of Right Wrist Three Views Taken on 10/26/22 Mottling of the carpal bones, with significant narrowing of the lunate fossa with loss of joint space, sclerosis, as well as continued atrophy of the carpal bones themselves. Previous ulnar styloid nonunion is still present. There is a narrowing of the midcarpal joint as well. These X-rays are consistent with previous images. No acute bony process noted. However, X-rays are concerning for progressive, localized osteoporosis and bone atrophy.

Upon return to the clinic, the patient reported his pain had resolved and his quality of life had returned to near normal, able to begin weight lifting again. However, the stiffness was still present. He completed the prednisone and was still taking the high-dose vitamins, as well, but ceased the gabapentin due to his pain resolving. He stated OT was especially useful in helping relieve discomfort. Updated three views X-rays of the right wrist (Figure 4) taken at this visit continued to demonstrate mottling of the proximal and distal carpal rows, including the radiocarpal joint and midcarpal joint. There appears to be an improvement of the bone morphology although continued significant loss of joint space in the radiocarpal joint and midcarpal joint. He was initiated on the range of motion brace to assist with stiffness. The patient was recommended to continue with hand exercises at therapy and at home. He was advised that there was the potential for chronic debility of his wrist regarding the joint space narrowing and loss of cartilage, as well as the likelihood that the bone morphology would not reconstitute itself. He was recommended to follow up in eight weeks for a hopeful release to activity as tolerated, leading to diagnosis of CRPS of the hand and wrist. He was aware, grateful, and functionally doing well, despite such horrific changes on plain film, MRI, and CT scan.

Upon his next follow-up, the patient reported significant improvement in pain and grip strength. The patient had completed formal outpatient therapy and was also participating in at-home therapy exercises. The patient has resumed attending the gym, only noting difficulty with movements involving marked wrist flexion. Excess hand hair growth has ceased, however, there was some residual hair on the dorsal aspect of the right hand. He did not have to shave his hand a second time. On physical examination, the patient noted minimal tenderness over the radiocarpal joint with continued stiffness. The patient had no hypersensitivity to touch, no TFCC pain, and no forearm pain. There was full composite flexion of the digits. The Arc of motion was 30 degrees of wrist extension to 25 degrees of flexion. Stiffness in wrist abduction and adduction was still significant. X-rays continue to show atrophy and changing morphology of the midcarpal joint and radiocarpal joint, with loss of space of the lunate fossa, as well as the articulation of the capital lunate joint. The bone demineralization was consistent with CRPS.

The patient was tolerating this condition fairly well, and was able to work without restrictions. Based on the affected wrist, his chronic wrist stiffness, his prognosis, and his young age, there is concern that this will be a chronic problem. There is a possibility that he will require future therapy, steroid injections, possible further imaging, and office visits, as well as ultimately requiring wrist surgery including wrist denervation surgery, spinal cord stimulation, and sympathetic nerve blocks. He was advised to continue the range of motion static progressive brace for flare-ups in stiffness. He was at maximal medical improvement (MMI) on this final visit. And, in spite of his being a work comp injury, he went back to work full duty, which was a wonderful thing in the setting of such a morbid work-related injury itself.

## Discussion

**Figure 5:**
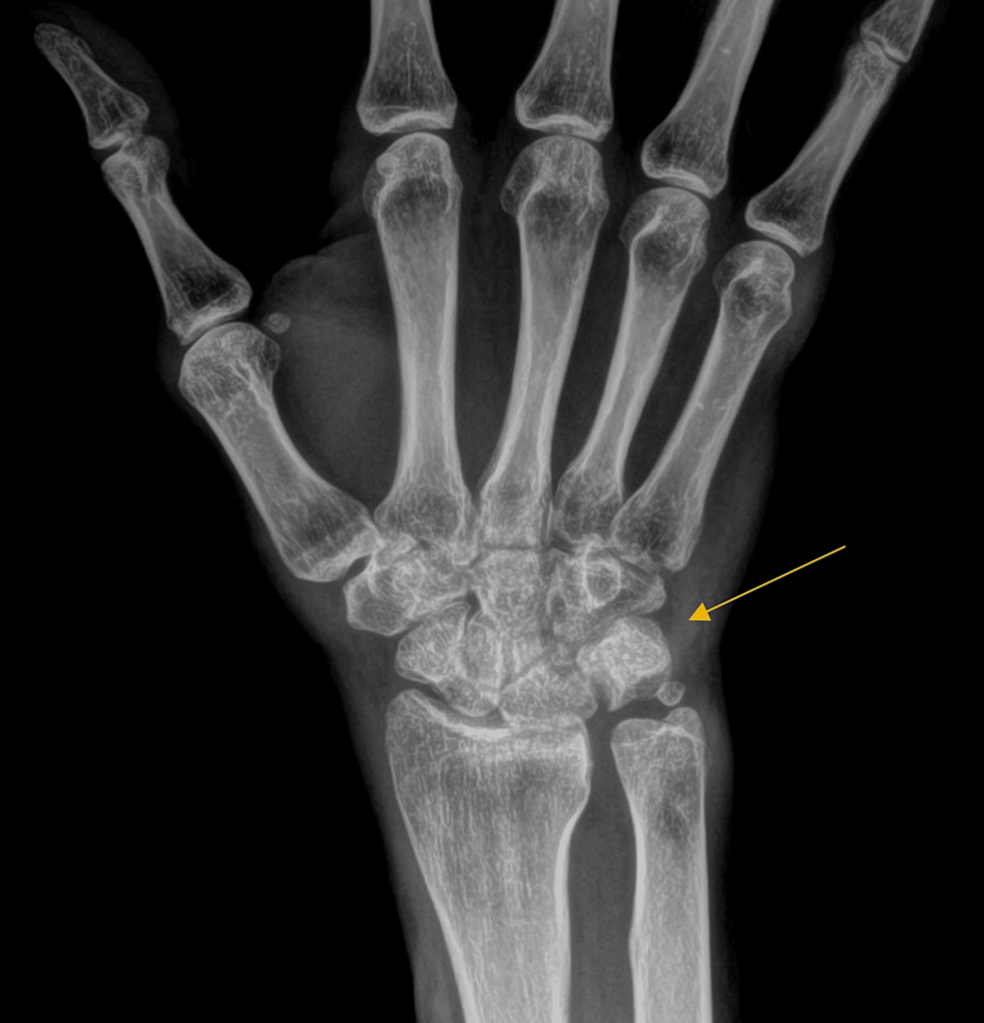
Final X-ray of the Right Wrist Taken on 10/26/2022 Gross demineralization and mottling of the carpal bones and arthritic changes of all surrounding metacarpals months after the initial date of injury.

We present a patient that we believe is a classic case of CRPS type 1. An insidious onset of increasing hyperalgesia, bone demineralization, gross skin and hair changes, worsening stiffness, and delayed healing led us to the diagnosis. The variable presentation, taxonomy, and treatment protocols associated with CRPS can cause confusion among physicians when encountering patients that we suspect may be expressing signs and symptoms of the disorder. Part of the challenge of diagnosing CRPS is the inconsistent use of diagnostic criteria among providers. The most recent criteria are the IASP-approved “Budapest” criteria which have four points to consider including continuing pain that is disproportionate to inciting event, report of two symptoms within several categories such as vasomotor and sensory, evaluation of three or more signs in the same categories, as well as the exclusion of all other possibilities [3]. Overlap with other rheumatic, inflammatory, or psychological conditions means that it is helpful to rule these conditions out if there is suspicion of CRPS [3]. Imaging criteria are not included in any diagnostic criteria that have been described for CRPS [4]. All imaging was done and helped exclude other causes of the patient's presentation. We utilized X-rays (which were initially negative/normal despite the presence of an ulnar ossicle variant [5]), computed tomography without IV contrast, and magnetic resonance imaging in the workup of this patient's described presentation, in addition to the clinical exam, a thorough history, and high level of clinical suspicion. Although these imaging modalities lack specificity compared to other modalities such as bone scintigraphy, which maps increased metabolism of the bone, we believe we were able to come to a diagnosis due to the combination of clinical findings and rapid onset of localized osteoporosis that was obvious in the other imaging studies. The last X-rays performed demonstrated a halt of progressive bone necrosis but were still consistent with gross changes in bone morphology (Figure 5) [4].

The patient has had a return to daily life despite gross mottling of the right carpus as a result of the multidisciplinary approach we used to manage the various symptoms that presented. Many different treatment protocols have been compared in the literature to give physicians a better idea of how to manage CRPS [6]. Our protocol consisted of aggressive corticosteroid usage, anticonvulsants, nonsteroidal anti-inflammatory drugs (NSAIDs), opioid analgesics, and other neuroprotective supplements, as well as edema gloves and desensitization. Our medical management was also supplemented with aggressive hand therapy which has been referenced multiple times in the literature to help decrease pain and increase the range of motion in the patient's wrist [7]. Results with each component within our protocol have shown variable responses when used separately [8-10]. Bisphosphonate therapy was considered and it has been used in several past cases, however, our patient was not keen on adding more medications and we were beginning to see improvement in his pain and stiffness prior to the addition of another drug [11]. Fortunately, this patient had no need for further invasive treatment which could have included peripheral nerve blocks, spinal cord stimulation, and trigger point injections [11]. An oversight that was made clinically was the initiation of both a COX-2 inhibitor (celecoxib) and steroids which have contradictory effects on bone healing in rheumatic conditions, however, our goal was aggressive inflammation control [12]. The extensive combination of anti-inflammatory, neuropathic agents, vitamins, prednisone, and opioids have shown a very positive response from our patient with the elimination of pain and return of quality of life as compared to the alternative of continued progression of the disease. Some mild bone re-mineralization was even exhibited towards the end of the clinical course.

## Conclusions

Complex regional pain syndrome is a rare disorder following a traumatic incident that can have a highly variable outcome depending on the severity of injury, category of the diagnosis, and treatment plan. Diagnoses can be typically achieved through an extensive clinical examination and in this case further imaging. We attest to an integrative approach in managing CRPS including but not limited to aggressive desensitization, physical/occupational therapy, anti-inflammatories, prednisone, nerve pain medication, and high-dose vitamin supplements including B6 and C. It is important for providers to communicate early about the working diagnosis, implement an aggressive game plan, and understand the variability in the presentation to allow for broad-spectrum treatment that will yield a higher quality of life in patients with this disorder.
